# When Should the Police Investigate Cases of Non-recent Child Sexual Abuse?

**DOI:** 10.1080/0731129X.2019.1600288

**Published:** 2019-07-02

**Authors:** Hannah Maslen, Colin Paine

**Keywords:** child sexual abuse, child sexual exploitation, non-recent offences, police ethics, Oxford CSA Framework, police investigation

## Abstract

Non-recent child sexual abuse (CSA) and child sexual exploitation (CSE) have received recent attention. Victims often do not report their ordeal at the time the incident occurred, and it is increasingly common for agencies to refer concerns to the police years, or decades, after the event. The combination of the non-recent nature of the offence, the lack of engagement by the (potentially vulnerable) victim, and the huge resource burden of investigation make deciding whether to proceed with investigation complex and ethically challenging. Although there will always be a presumption in favor of investigation, for some cases the reasons against investigating will outweigh this presumption. We examine the considerations at stake in making a decision about whether to make contact with the victim and proceed with investigating a particular non-recent CSA case. Arguing for a “broad rights” approach, we identify considerations relating to (1) the victim, (2) criminal justice and crime prevention, (3) limited resources, and (4) legitimacy. We argue that, all other things being equal, non-recent and current investigations are equally worthy of investigation. We assess the implications of suspects being persons of public prominence. We outline a principled decision-making framework to aid investigators. The Oxford CSA Framework has the potential to reduce unnecessary demand on police resources.

## I. Introduction

Non-recent child sexual abuse (CSA) has received increased attention in recent years in the United Kingdom and elsewhere. Complaints against various public figures have attracted significant media attention^1^ through Operations Yewtree,^2^ Midland,^3^ and Conifer,^4^ among others. Operation Yewtree in particular focused attention on non-recent CSA investigations. Operation Yewtree has been led by the Metropolitan police service since 2012 in response to sexual abuse complaints, predominantly the abuse of children, against the British media personality Jimmy Savile and others. Child Sexual Exploitation (CSE) cases in places such as Telford,^5^ Rotherham,^6^ Rochdale,^7^ and Oxford^8^ have further increased public awareness of the problem. CSA involves forcing or enticing a child or young person to take part in sexual activities. CSE is a subcategory of CSA and occurs where a child is persuaded, coerced, or forced into sexual activity in exchange for, amongst other things, money, drugs or alcohol, gifts, affection or status.

The police service continues to see an upward trend in the number of reports of non-recent CSA, where non-recent is defined by Operation Hydrant^9^ as meaning that the abuse ended at least one year prior to reporting it to the Police. The Office for National Statistics’ (ONS) Crime Survey for England and Wales estimated 567,000 women and 102,000 men were victims of rape or sexual assault as a child; 7% of all those surveyed had suffered sexual abuse as a child.^10^ The ONS found that three quarters of adults who reported having experienced CSA had not told anyone. Non-recent offending represents a significant proportion of all CSA reported to the police; 38% of all recorded sexual offences against children are reported to the police one year or more after the offence took place.^11^

The harm caused to childhood victims of CSA is significant and often enduring. Being a victim of CSA is associated with an increased risk of adverse outcomes in all areas of life. This includes harms to mental health, physical health, intimate relationships, educational attainment and vulnerability to further revictimisation.^12^ Victims are not a homogenous group, and the extent and manifestation of these harms varies significantly. However, sexually abused children often find it difficult to report their ordeal at the time the incident occurred. It is common for other agencies to refer concerns to the police years, or even decades, after the event.^13^

The non-recent nature of the offence combined with third-party reporting presents investigators with a challenging ethical dilemma when making the decision about whether to proceed with investigating a particular case; specifically, the decsion about whether to make initial contact with the victim, which would be necessary to conduct a full investigation. Since the victim has not come forward, it is unknown whether they want an investigation to be pursued. Further, particularly vulnerable victims may be at risk of psychological harm were the police even to approach them regarding their past ordeal. In addition, such investigations can be hugely resource intensive. However, the gravity of the offence and the potential ongoing threat posed by the suspected offender are substantial considerations weighing in favor of proceeding with the investigation.

Existing police decison-making resources, such as the National Decision-Making Model and the Code of Ethics, are well suited to day-to-day decision-making but are not intended to address the intricacies of specific strategic decisions. Although guidance on investiging CSE cases, including the College of Policing’s “Authorized Professional Practice on Responding to Child Sexual Exploitation” and “Operation Hydrant SIO Advice”, details *how* investigations should be conducted, it does not advise how to determine *whether* they should be conducted. Whilst we would expect good decsions not to be in conflict with estabilished guiding principles, these principles alone do not resolve what to do when there are multiple ethical considerations. Although deciding whether to investigate a non-recent CSA case raises some issues also present when deciding whether to investigate domestic abuse cases involving an unwilling victim, CSA decsions are in other ways unique: the combination of the age of the victim at the time of the offence, the substantial resource requirement, and the particular risk to the victims’ psychological stability generates a need to rethink the ethical and strategic justifications for investigational decisions.

In this paper we examine the considerations at stake in making a decision about whether to investigate a particular CSA case. We begin by emphasizing why the decision about whether to investigate or not is in large part an ethical decision, and why careful analysis will add clarity. We then situate our discussion against the background of existing literature on normative theories of policing, explaining why they, and the existing police decision-making models, do not provide a comprehensive answer to the particular dilemma generated by non-recent CSA cases. Next, we set out the considerations at stake in the decision, distinguishing between those that relate to (1) the victim, (2) criminal justice and crime prevention, (3) limited resources, and (4) legitimacy and perception of the police, including public interest in complaints against people of public prominence and institutions. We argue for an overlapping hierarchy of these ethical considerations, based on the relative strength of the moral reasons they generate. Although the considerations somewhat overlap, in general some of them generate stronger reasons for or against investigation than others. Drawing on this analysis, we set out a principled decision-making framework to aid investigators.

## II. The Need for Guidance for Non-recent Child Abuse Investigation Decision-making

Before outlining why guidance for decision-making is needed, we must clarify the circumstances in which investigators will be faced with making a decision. Not all cases will require the investigator to consider whether or not to proceed with investigation. For instance, cases normally will be investigated if any of the following apply:

The victim reports the offence and asks the police to investigate.
The victim, or other victims, appear to still be at immediate risk of harm from the offender.
The police know or have reason to believe the suspected offender is a current threat.

In contrast, there are some common features to cases that require a decision about whether to investigate. They typically involve offences that occurred some time ago, and the suspected victims have usually *not* reported the offence to the Police; the victims are typically now adults, who often seem to have “moved on” with their lives. The potential offences have come to the attention of the police via third parties, such as other agencies. They also typically involve suspected offenders who appear not to have continued to offend, or who are unable to offend because they are in prison or deceased. In such circumstances, the principal question investigators face is whether or not to make the first approach to the suspected victim.

Whilst we shall suggest that there will nearly always be reasons to investigate (generating a defeasible presumption to investigate), this does not eliminate any reasons present that weigh against investigating. These competing reasons generate a complex ethical dilemma for investigators, the resolution of which requires a principled decision-making approach. This difficulty is exacerbated by the unavoidable uncertainty surrounding outcomes. As we elaborate below, existing resources and normative theories do not provide sufficient guidance.

## III. Normative Theories of Policing and Existing Guidance

The ethics of policing has received both academic and professional attention. Despite this fact, the existing work is insufficiently directive regarding the particular issue of investigating non-recent CSA offences. Existing normative theories of policing provide general justifications for policing practice and some focussed guidance on specific policing activities. Whilst general principles may be applicable in the present decision-making context, we will show that further fine-tuning is required. Existing professional frameworks developed for policing are insufficiently nuanced or ill-suited for the dilemmas that require resolution. In this section, we briefly outline existing normative theories of policing and professional guidelines, emphasizing their strengths, but explaining why they do not provide all the tools needed to make decisions about these cases.

### 1. Normative Theories of Policing: Defending a “Broad Rights” Approach

Seumas Miller and colleagues have developed a particularly comprehensive normative theory of policing, and have applied this theory to criminal investigation.^14^ Miller’s theory is rights-based and “teleological.” Its primary purpose is to explain why policing, involving the significant use of force and other infringements of liberty, is justified. Miller claims that policing is justified (and that its aims are structured) by its purpose to protect legally enshrined, justifiably enforceable, moral rights—rights to life, property, security and so on. Miller and Blackler summarize their theory as follows:

In short, in our view police ought to act principally to protect certain moral rights, those moral rights ought to be enshrined in the law, and the law ought to reflect the will of the community. Should any of these conditions fail to obtain, then there will be problems. If the law and objective (justifiably enforceable) moral rights come apart, or if the law and the will of the community come apart, or if objective moral rights and the will of the community come apart, then the police may well be faced with moral dilemmas. We do not believe that there are neat and easy solutions to all such problems.^15^

Miller and Blackler’s theory is nuanced and persuasive. They identify the protection of moral rights (both human rights and institutional rights) as the principal purpose of policing, constrained by democratically supported laws. They acknowledge that even with this normative theory in place, ethical dilemmas may arise. In part, as they suggest, this could be a result of a lack of coherence between the justified protection of moral rights on the one hand and the will of the community on the other. In the case at hand, the will of the community might favor investigating all and any leads regarding CSA, whilst the victim’s (defeasible) moral right to privacy might be significantly infringed by persistent, unwanted investigation.

However, we suggest that whilst a theory such as Miller’s provides a plausible high-level justification of policing, not all of the ethically relevant considerations in CSA decision-making are captured by enumerating the legally enshrined moral rights protected by one course of action versus another. The moral relevance of some considerations is best captured by focussing directly on the “rights-like” interests at stake, beyond those that are legally enshrined. Crucially, these considerations may fall outside the scope of “established” moral rights; for example, although individuals have a right to privacy, there is not an established right “not to be caused significant *mental* harm.”^16^ Yet, interests in mental and social stability can generate moral reasons because they are fundamental to agents’ self-governance and well-being, even if they do not ground a legally enshrined right.

Extending the scope of the relevant considerations to include interests that are fundamental to agents’ self-governance and well-being does not commit us to taking a consequentialist approach, with all its attendant shortcomings. Whilst we cannot here take up the debate regarding what grounds a moral right and which moral rights we hold, we suggest that there are some interests agents have in non-interference that generate moral reasons that are potentially weaker than those underlying established moral rights, but which nonetheless plausibly ground defeasible moral duties and obligations. The interests we have in mind will generate defeasible constraints only on certain types of particularly harmful or intrusive interference, rather than opening the door to a crudely consequentialist moral calculus.

Giving weight to those of the victim’s interests that are fundamental to their self-governance and well-being still allows for the possibility that certain other considerations might be sufficient to outweigh them. However, it also lends support to the claim that these other considerations will have to be particularly weighty if they are to be sufficient to do so, given the moral salience of the victim’s interests. Our approach therefore accepts that the protection of moral rights principally directs (and justifies) the activities of the police, but contends that there are additional “rights-like” considerations in the CSA context, generated by individuals’ interests in not being subjected to interventions that severely compromise their mental and social stability. These “rights-like” considerations are grounded in respect for individuals and their ability to function as agents.

Beyond direct protection of moral rights, considerations of distributive and penal justice, and the democratic legitimacy of the police generate additional considerations that are relevant to pursuing the protection of legally enforceable moral rights, without prompting a shift to a consequentialist assessment of net utility. More or fewer individuals’ rights will be protected depending on where resources are directed, and so the moral significance of limited resources concerns fair distribution of rights protection and non-violation. In relation to democratic legitimacy, Miller highlights the relevance of the “will of the community” to the protection of rights (whilst not claiming that policing is simply a matter of fulfilling that will). Our approach might therefore be seen as the deontological counterpart of “constrained consequentialist” approaches in public health and criminal justice.^17^ Whilst those approaches justify compulsory interventions (such as quarantine in the context of infectious disease) as long as certain rights are not unnecessarily or disproportionally violated, our “broad rights” approach takes into account (i) “rights-like” interests that are fundamental to agents’ self-governance and well-being, which generate defeasible negative obligations even if these are not currently granted legal protection, (ii) positive claims to *just* distribution of rights protections and public resources, (iii) the democratic relevance of societal endorsement for organizational legitimacy, and (iv) any inherent value in dispensing justice.

This is not a criticism of Miller’s theory. Rather, we have demonstrated that there will be some supplementary considerations in specific decision-making contexts. Indeed, Miller and Blackler say that their theory is “not a theory about specific police methods or strategies; it is not a theory of, so to speak, best practice in policing.”^18^ In the remainder of this paper, we provide arguments regarding best practice in non-recent CSA investigation, given the ethical dilemmas such investigations can pose. Following some comments on the existing guidance available to investigators, we approach the dilemma by setting out all the relevant considerations.

### 2. The National Decision Model, the Code of Ethics, and Other Sources of Guidance

The police service of England and Wales has adopted a National Decision Model (NDM).^19^ The model, which has six key elements, is considered to be suitable for all decisions. Decision-makers can use the NDM to structure a decision-making rationale. Arguably, investigators could simply use the NDM in making decisions about the investigation of non-recent CSA. However, the model does not easily facilitate decision-makers to trade off threats against one another or to weigh two or more different types of threat.

The NDM has the Code of Ethics at its center and aims to put ethics at the heart of decision-making.^20^ This encourages all decisions to be consistent with the nine principles and ten standards set out in the Code. However, the standards simply set out the behaviors expected of officers, such as honesty and integrity. They perhaps most usefully identify something like virtues for officers to cultivate, without being action-guiding in specific contexts. The nine principles in the Code of Ethics are derived from the Nolan Principles,^21^ and include accountability, objectivity, and selflessness, for example. It is difficult to see how such principles, on their own, could help an investigator to make the very best ethical decision as to whether to visit a potential victim of non-recent CSA. This is not a criticism of the Code, rather a recognition that it should not be made to do work it was not designed to do.

Other existing authorized professional policing guidance on the investigation of non-recent CSA recognizes the range of factors that impact on the potential for harm through investigation, but does not go on to identify the relative weight of each of the considerations, nor how they might be considered or traded off against each other to form an ethically nuanced view on the merits of investigation, leaving investigators to undertake a form of artistry in forming their policy decisions.^22^ This may lead to investigators reaching differing conclusions when confronted with ethically identical cases.

## IV. Relevant Ethical Considerations and the Presumption in Favor of Investigation

There is plausibly a *prima facie* presumption towards investigating suspected cases of non-recent CSA, a presumption that is principally grounded by the value of justice, and the general deterrent effects anticipated by successful investigation, ultimately protecting moral rights. These considerations generate a presumption since they will apply to all cases and will be mostly consistent in normative weight. This presumption must be outweighed by countervailing reasons if an investigation into non-recent CSA is not to proceed; we shall suggest that there are cases where this presumption can be defeated. Moreover, the presumption itself, as we shall argue, can be tempered or moderated by certain factors relating to victim privacy and the likelihood of successful prosecution.

This presumption and the following considerations set the foundation of the ethical framework we propose in the remainder of the paper; in applying the framework to a particular case, one must consider how these factors vary and may exert different relative strength from case to case. In arguing for the CSA framework, we demonstate not only that there are multiple moral consideratons relevant to the issue, but that there are types of consideration that are particuarly relevant in this context, but which may not be so relevant for other types of case. Whilst our *approach* could be used as a model for how to think through what is at stake in any given decision, the types and weights of the relevant considerations depends on the details. As such, our framework is not intended to be a one-size-fits-all set of principles that can be straightforwardly applied outside the CSA context.

We first set out the nature and significance of the victim-centred reasons that weigh against the presumption, before turning to the reasons that weigh in its favor. We consider how limited resources intersect with achieving policing purposes, requiring prioritization of those activities that better serve the goods pursued in policing. We then consider reasons to investigate grounded in public confidence. Although we will sometimes refer to “harms” in what follows, the harms we invoke involve rights and “rights-like” violations, in keeping with our broad rights approach. Similarly, we sometimes refer to rights protections, justice, and legitimacy considerations as “goods.”

### 1. Considerations Relating to the Victim

#### a. Respect for Victims’ Wishes and their Privacy

There is a range of potential explanations for a victim’s decision not to report their ordeal. Shame, guilt and embarrassment, together with concerns about confidentiality and fear of not being believed are prominent.^23^ Indeed, many victims are not even sure that the incidents are real crimes, due to cultural messages that trivialize certain crimes.^24^ In some cases adaptive indifference, an adaptive response to conflicting norms and allegiances may discourage victims from reporting misconduct. Whilst it will be difficult to know why a *particular* victim has not reported the offence to the police, respect for the victim’s wishes and their privacy generate moral reasons that in some cases point away from investigation.^25^ Understandably, many victims have no wish to relive the offence. Initiating an unsolicited investigation might then significantly frustrate the victim’s wishes. Indeed, we might think that the nature of these particular offences—involving significant coercion or compulsion—makes consideration of victims’ wishes particularly important. This consideration generates a reason not to investigate, which we will argue tempers the presumption where solvability is low.

There is some evidence to suggest that when victims do not come forward, it is more likely that they do not want an investigation.^26^ Victims have plenty of opportunity to approach the police and request an investigation without the need for police to pro-actively approach them; moreover, numerous recent high profile cases of non-recent CSA, such as Operation Yewtree, have prompted other victims to come forward themselves. Further, some evidence suggests that when police pro-actively approach potential victims of non-recent offences, they are unlikely to engage in the investigation; in a recent investigation approach to over two hundred potential victims, just 10% chose to engage.^27^ Given that investigators cannot know for sure what a particular individual might want, there are reasons to assume that initiating an investigation will involve unwanted intrusion.

Frustrating a victim’s interest in privacy is not the only interest of the victim at stake, as we indicated above. A victim might plausibly experience substantial psychological harm from the police initiating an unwanted investigation. These interests may interact and overlap. However, it is important to separate them, as some victims may be much more vulnerable to psychological and social harms of investigation than others.

#### b. Victim Well-being: “Rights-like” Interests in Mental and Social Stability

An investigation can bring about “secondary victimization,” which is defined as treatment that exacerbates the trauma of the initial assault.^28^ Contact with the criminal justice system can be revictimizing; for example, victims may be asked about their sexual histories, what they were wearing and how they behaved at the time. Victims report that such interactions can be highly distressing and leave them feeling guilty, depressed, anxious, distrustful, and reluctant to seek further help after interacting with the criminal justice system.^29^ If there is information that suggests that the victim is vulnerable, would experience significant psychological distress, social stigma, or significant upheaval to their life, this generates a strong reason not to investigate. Recent investigations of complex CSE are replete with examples of adult victims’ relationships failing or victims self harming after an unsolicited visit from the police seeking to conduct an investigation many years or decades after the event. Indeed one phrase frequently directed at investigators is “my life was okay again until you lot came along.”^30^ Mounting evidence of this sort has prompted a rethinking of the ethics of proceeding with investigations where psychological harm is likely to be high.

A thorough partnership risk-assessment of the victim’s mental and physical health, and his or her safety, is already established practice, and informs the assessment of how much harm investigation might do. When a decision is made to visit a victim, victim support and counseling are often at the heart of the investigative strategy. Victims will be supported through the criminal justice process and may be entitled to enhanced support and special measures.^31^ However, even with a clear strategy to minimize or mitigate any harm to the victim, such trauma cannot be precluded.^32^ Further, well-being considerations will often extend beyond the immediate victim: there are risks posed to close family and friends of the victim; marriages can break down, and children can be affected by the trauma of a parent.

The strength of the moral reasons generated by consideration of the victim’s well-being will vary, depending on how significantly and how likely it is that they will be harmed. We argue below in section 6 that these reasons, although not decisive, are w*eighty enough to tip the balance towards not investigating in some cases*.

### 2. Considerations Relating to the Purposes of Policing: Crime Prevention and Criminal Justice

We now outline the most significant reasons supportinging investigation, generated by the goods that policing facilitates or achieves—goods that justify and legitimize the practice of policing, in keeping with our broad rights approach.^33^ Some of these, as noted, ground the presumption to investigate, since they are present in all cases. Others generate additional reasons to investigate in particular cases.

#### a. Crime Prevention: Incapacitation and Deterrence

A central purpose of policing is to protect citizens, including through crime prevention. As noted above, Miller argues that policing is justified by its purpose to protect legally enshrined, justifiably enforceable, moral rights, including the rights to life and bodily integrity. Investigation prevents harm (often rights violations) when it leads to conviction and criminal punishment of offenders who would have reoffended, and deters would-be offenders.

The most direct method of harm prevention is the *incapacitation of offenders* who are likely to reoffend. Sentences for CSA are severe.^34^ Where investigation leads to incarceration of an offender who would have reoffended, it directly serves a harm-prevention purpose by preventing the offender from causing harm for the duration of his or her sentence, and with enhanced safeguards after their release (such as being subject to multi-agency public protection arrangements). We should be careful not to assume that all offenders are likely to reoffend, however. Contrary to the widely held perception that the risk posed by sexual offending is “high, stable and linear,”^35^ the reality is that recidivism rates for these types of crimes are relatively low in comparison to other types of offending.^36^ Meta-analysis suggests a recidivism rate of 13.7% after five years; however, these observed rates are likely to be an underestimate due to the underreporting of sexual offences. Nevertheless, the recidivism rate for sexual offenders is lower than for the offending population in general. It is worth noting, though, that a small subset of sexual offenders has a much *higher* rate of re-offending—these offenders pose the threat with which we are concerned, and intelligence can assist with determining whether an offender is likely to fall into this subset by identifying risk factors.^37^

The strength of the reason that the prospect of incapacitating the offender generates will depend on the level of threat that offender poses: how likely that particular offender is to reoffend and how serious those offences are likely to be. In making the judgement in relation to harm prevention, officers must consider the potential for the offender to harm others, perhaps not yet identified, as well as the known victim. In making this judgement there is of course a considerable degree of uncertainty. It is perhaps for this reason that the Authorized Professional Practice (APP) Risk Principle 1 states that “The willingness to make decisions in conditions of uncertainty (i.e. risk taking) is a core professional requirement of all members of the police service.”^38^ Information or intelligence relating to the level of threat the suspected offender poses, including assessment of probability, is therefore critically relevant to the strength of the reason that offender threat generates.

Conviction and punishment can also prevent harm by *deterring the offender and/or other potential offenders.* These two effects are often referred to as specific (individual) and general deterrence, respectively.^39^ Deterrence occurs when an individual chooses to comply with the law through fear of the consequences of not doing so. Research indicates that the criminal justice system exerts a powerful deterrent effect on would-be offenders. The extent of this effect is dependent upon the certainty and speed of apprehension, and the severity of any subsequent sanction. However the certainty of apprehension has a greater deterrent effect than the severity of any subsequent punishment.^40^ The investigation of a CSA offence (and conviction) will have deterrent effects, both on the suspected offender and on potential offenders who learn of the investigation.

The strength of the reason to investigate generated by the prospect of *specific deterrence* will again be related to the threat the offender poses, and how likely it is that deterrence will operate to reduce this threat. Although this likelihood will vary between cases, we suggest that any theoretical relevance of specific deterrence is in practice eclipsed by the far more certain and substantial harm-prevention effects of incapacitation through incarceration. This is because incapacitation is a certain way to prevent harm, and the estimation of any prospects for specific deterrence relate to a time far in the future, given the duration of the custodial sentences imposed for CSA offences. Specific deterrence will, in practice, not bear on the decsion.

In contrast, the effects of *general deterrence* on harm prevention, although hard to calculate, are more weighty, given that many would-be offenders are not incarcerated. Indeed, we argue that the reasons generated by general deterrence, partly ground the initial presumption to investigate. Since the prospect of punishment serves a general deterrent purpose, it provides a *pro tanto* reason to investigate in all cases. *Investigators do not, therefore have to further consider the general deterrent effects expected for a particular case, since these already ground the presumption*, which must be outweighed if investigation is not to go ahead. In contrast, the variable threat posed by the offender, which could be elimitated via incarceration, is not incorporated into the presumption, and must be considered separately.

#### b. Justice: Desert and Expression of Censure

Police investigation indirectly serves criminal justice and is therefore an extended aspect of policing purpose. Through conviction, the state communicates censure to the offender and publicly denounces their conduct. This is independent from any harm prevention resulting from incapacitation or deterrence.

The view that retribution and expression of censure provide a sufficient justification for punishment (independently of any consequences for crime prevention) is contested.^41^ The question of how much weight to place on any reasons generated by retributive justice will be similarly contested. However, it is not controversial to claim that punishment serves an important expressive purpose, even if this is understood in less strictly retributive terms, along the lines of reinforcing the norms of society and communicating appropriate disapprobation.^42^ Independently from any deterrent effects, the state uses conviction and punishment to express justified condemnation of the proscribed conduct to both the offender and citizens.

We suggest that, along with general deterrence, the reasons generated by considerations of justice partly ground the presumption to investigate: it is always of value that serious wrongdoing is acknowledged and condemned, and the police play an important role in bringing this about through investigation. If, as we have suggested, this consideration is broadly uniform across offences of a similar type, it would follow that investigators would not need to further consider it in their decision-making, since it is already accounted for in the weight of the presumption.

One might argue that a strain of retributive thought might speak against investigating some instances of non-recent CSA. If one maintains that the culpability of offenders diminishes over time, then considerations of justice will speak less strongly in favor of investigating historic crimes. We reject this possible line of argument. Culpability—or blameworthiness—is a function of one’s moral responsibility for the offence: how much one controlled and intended what happened, fine-tuned by any mitigating or aggravating factors.^43^ It is not possible for the offender to retroactively change what happened at that past time, nor their contribution to it. Culpability for the past event itself therefore cannot diminish over time.^44^ However, an individual’s culpability may be aggravated or supplemented over time, due to their failure to voluntarily admit to their wrong doing in the intervening period.

A related argument, that the censure due to the offender—the appropriate formal response—diminishes over time is also unconvincing.^45^ The offender, having evaded prosecution, may have come to believe that their conduct was not as blameworthy as it was. This may also be true for anyone who knew about the offences, including the victim. There is therefore good reason to counteract this impression by condemning the criminal act just as harshly.

Rather than the non-recent nature of the offence diminishing the offender’s culpability, we suggest that the intuition that culpability is reduced can be debunked. The intuition is more plausibly understood to be an unwarranted inference from more defensible claims relating to (1) the diminished *solvability* of non-recent cases, and (2) a mistaken assumption that offender *dangerousness* tracks the recency of their crimes, which, if not mistaken, would be relevant not to justice but to crime prevention.

In relation to solvability: the investigation of crime becomes more difficult as time passes because of the attrition of evidence; documents are lost, CCTV is wiped, and memories fade.^46^ The investigation of non-recent offences is thus typically more challenging and more resource-intensive for the police.^47^ These practical difficulties may be mistakenly conflated with the justice value of conducting an investigation into a non-recent offence. The practical challenges are morally relevant in their own right, and, as we argue, will temper the presumption to investigate to some extent, but this is only contingent on non-recency.

In relation to dangerousness: it might be also assumed that the threat posed by non-recent offenders is low, such that they are unlikely to pose a risk of future harm. However, recent cases have shown that some offenders have long offending careers spanning decades before being caught and therefore the intuition that all non-recent cases pose reduced risk is incorrect.^48^ Regardless of whether this applies in every case, and how it therefore impacts reasons generated by offender threat, we have argued that the good that investigation could achieve in terms of justice is unaffected by time. Accordingly, reasons to investigate generated by considerations of justice are equally as strong for non-recent cases as they are for present cases with the same features.

### 3. Considerations Relating to Limited Resources

The investigation of complex CSE cases is particularly resource-intensive. Operation Stovewood, the investigation into CSE in Rotherham, is a particularly striking example with potentially 426 suspects involved in offending against up to 1500 victims with a potential cost of up to £90 m.^49^ Analysis within Thames Valley, UK suggests that the average complex CSE case has seven victims, eighty-eight witnesses, twenty-one suspects and ultimately ten defendants.^50^ On average a complex CSE investigation^51^ takes nine investigators two years to complete and will cost £885,140 to resource from start to finish. These cases arise with a remarkable degree of regularity, arising on average every six months in Thames Valley alone.

The resourcing challenge is particularly acute in the current climate of austerity. Police officer numbers have been reduced by over twenty thousand since 2007/08, a 16% drop, and police numbers are now at the lowest levels since 1981^52^ prompting many Chief Constables to speak out about the necessity to “ration” investigations.^53^ This challenge is further accentuated by the difficulty many forces are facing in the recruitment of adequate numbers of detectives; leading HM Inspectorate of Constabulary and Fire & Rescue Services (HMICFRS) to estimate a national shortfall of five thousand detectives.^54^

This context of limited resources raises ethical questions about prioritization: some things inevitably will be done less well, or not at all. Reasons not to investigate non-recent CSA are generated by these considerations when significantly more of the relevant goods of policing can be achieved were the resources directed elsewhere, to prevention or investigation of other serious offences, for example. The relevant goods are those identified above, underlying and related to policing purpose. Limited resources matter ethically here because they force a determination of how to *best or most* fulfill policing purpose, when it is not possible to fulfill policing purpose maximally. This is not equivalent to maximizing utility: distributive justice bears on the fair distribution of resources and rights protections, particularly when investigation of other serious offences would be deleteriously affected.

Decision-makers must consider the resources that an investigation is likely to require, and how much directing resources to that investigation would compromise other policing priorities, such that the achievement of harm prevention and justice would be net-reduced or unfairly distributed. These opportunity costs are morally relevant. *Where there would be significant widespread impact on other activities, a reason is generated not to direct resources to the CSA investigation.* Recent analysis of serious sexual assault investigations in Thames Valley showed that the average investigation takes 77.3 h which means each officer could complete just 14.9 investigations in a year.^55^ Therefore, on average, a decision to investigate a complex non-recent case would be the equivalent of undertaking 268 serious sexual assault investigations; a significant resource commitment. This is not to suggest that it would be a direct choice between investigating the complex case or to investigate the 268 assaults (resource would be moved from lower priority crime types to carry out these investigations), but nevertheless it gives a sense of the scale of the commitment.

### 4. Police Legitimacy: Confidence in the Police and Promises Made

Trust in the police is critical to their effectiveness and their legimimacy.^56^ If decisions in CSA cases are perceived to be unfair, or to be contrary to community values, then this could pose a significant risk to public co-operation with the police and law-abiding attitudes.^57^ Therefore, the perceived fairness of such decisions needs to be carefully considered. These considerations will have a significant local context—for example, a recent failed investigation into CSA that attracted public concern may weigh in favor of conducting an investigation. Conversely, if local concerns about police resourcing were acute, then what could be perceived as unnecessary and illegitimate trawling for non-recent offences may weigh against conducting an investigation.

On occasion, police forces may make statements of commitment to the priority of CSA investigations.^58^ Such public statements may weigh in favor of conducting an investigation; particularly when an explicit promise has been made to the community that all such offences will be investigated. Failing to adhere to a public commitment could undermine the legitimacy of the police

#### a. Persons of Public Prominence and Institutions

In recent years, investigations into high profile public figures and institutions have been conducted. Some of these investigations have resulted in convictions, and others have resulted in reputational damage for the forces investigating. On occasion, the latter result has led to concerns that the police should not have undertaken the investigation or given credibility to those making complaints against people in the public sphere.^59^

Whilst the starting position should be that everyone is equal before the law, such cases do present unique challenges to investigators. One consideration raised by these cases is the potential for significant reputational damage and consequent psychological harm for those accused in the public eye.^60^ Recent high profile cases have shown the harm that can be caused in such cases, which is larger in scale than that caused to an ordinary member of the public owing to the greater media interest in such cases.^61^ Those in positions of public prominence may be at risk of becoming victims of false complaints, purely as a result of their celebrity, in a way that other members of the public are not. Recent examples of this type have seen complainants convicted of having made false complaints.^62^ Investigators in these cases are often placed in an invidious position. The fact that a complaint of serious crime has been made will usually warrant an investigation, but the act of investigating can cause considerable harm to those under investigation if complaints turn out to be spurious.

This said, deciding not to pursue an investigation into a high-profile figure may provoke claims of a cover up,^63^ or concerns that the police are not acting dispassionately. Further, investigations into complaints of CSA that have occurred in institutional settings may generate additional reasons to investigate, owing to the potential for the institution to have either been complicit in the abuse, or to have been negligent in failing to prevent the abuse. Exceptionally, then, such cases may generate an unusually strong reason to investigate for reasons of public interest, not only to prevent further such harm in the institutions, but also to enable broader societal learning regarding what went wrong.

Therefore, whilst it is true that everyone is equal before the law, the public prominence of an individual and the involvement of an institution creates distinctive considerations for investigators.

## V. Procedural Considerations and Police Accountability

Policing, and police officers, are rightly held to high standards of accountability. Such accountability can come years, sometimes decades, after the event, when memories of the decisions made and their rationale may have faded. The Independent Office of Police Complaints (IOPC)^64^ is charged with the investigation of alleged police misconduct, and can refer officers to gross misconduct hearings with the potential for officers to be dismissed. In some cases, poor decision-making by officers might be considered to meet the criminal threshold of manslaughter by gross negligence, or malfeasance in a public office.^65^

Such considerations can skew decision-making, leading to officers not aiming to arrive at the right decision all things considered, but at the decision that leads to the lowest risk of personal liability. The literature on police culture recognizes that “street cops” can consider that “management cops’” priority is to “cover their arses”; that is, undertaking activity to protect themselves from subsequent criticism.^66^ Ideally, any decision-making process should enable officers to arrive at a decision in the right way, and thereby provide ample subsequent justification for their decision-making.

This said, it is possible that despite the most careful consideration and weighing of all the relevant considerations, some serious harm could subsequently result. A good decision-making process cannot guarantee a good outcome, although it may increase its likelihood. However decision-makers must not be dissuaded from making such judgements through fear of subsequent criticism. Fear of mistakes can dissuade decision-makers from making the most appropriate decision on the information available to them. The fact that a good decision sometimes has a poor outcome does not mean that the decision itself was poor.^67^ Indeed, case law recognizes that courts will support reasonable and defensible risk taking in decisions.^68^

## VI. Implications for Decision-making Frameworks

The above considerations provide some reasons to investigate non-recent CSA and some reasons not to. In order to determine whether an investigation should be conducted, principled guidance is needed on the relative importance of these considerations, and how to weigh their significance in any particular case. In this section we argue for an overlapping hierarchy of considerations, and explain how features of the case will affect the strength of the reasons that the considerations generate, with implications for the most justified course of action. Having argued for this hierarchy, we outline a useable framework to guide decision-making, showing how it will support the decision-maker to reach different conclusions depending on the features of the case.^69^

To determine the correct hierarchy of considerations, we must focus on two things: (i) the inherent moral significance of the considerations and (ii) how much weight we should accord the reasons these considerations generate, given uncertainty regarding outcomes. The strength of a reason will roughly track the relative moral significance of the consideration, discounted for likelihood. However, a particular consideration can be so significant that it generates a strong reason, even if the likelihood of the relevant outcome is fairly low. Given that decisions are always made in the context of limited information and uncertainty, the decision-making process should prioritize limiting the potential for the most serious rights violations.

Although “rights-like” interests of potential victims could be significantly frustrated through unsolicited police approaches, we argue that the worst kind of error would be to fail to prevent further CSA. This is because (i) the sexual assault and exploitation of children involves the most egregious of the rights violations at stake in the decision, (ii) in addition to these rights violations, CSA victims are also likely to suffer *additional* psychological harms of the sort that undermine their “rights-like” interests in mental and social stability, and (iii) there are opportunities to provide past victims who are harmed by investigational intrusion with compensatory harm mitigation and support. In contrast, prevention of further CSA perpetrated by the offender cannot be similarly controlled without investigation. So, in assigning weighting to the reasons generated by the considerations, we aim to *reduce the likelihood of the worst kind(s) of error*, even if this means increasing the likelihood of errors of less severe moral significance. The weight of reasons to take a course of action that could lead to the worst type of error would need to be weightier than just tipping the balance.

### 1. Relative Significance of Considerations and Strength of Reasons Generated

As argued above, the goods—of general deterrence and justice—that successful investigation of CSA offences will uniformly achieve, generate a presumption to investigate. Before considering whether this presumtion can be outweighed, we note that the presumption itself can initially be weakened. The harms of (assumed unwanted) privacy violation generate a reason to temper this presumption when “solvability” (the likelihood of gathering sufficient evidence to convict an offender) is low.

Victims’ interests in privacy are not alone sufficient to temper the presumption. The moral significance of the rights violations the offender might go on to commit if not investigated may justifiably override the victim’s interest in privacy. Whilst this latter interest is substantial, the prevention of future sexual assaults (and asscoiacted psychological harm) plausibly carries greater weight, *where this is a sufficiently probable outcome of investigation leading to prosecution*. This is not to suggest that victims have a moral obligation to maximally and proactively engage with the police to initiate investigation—this would be too demanding. However, one can deny that this demanding obligation obtains whilst maintaining that the police would be justified in pursuing an investigation against the victim’s wishes, if considerations of harm prevention weigh in favor of investigation.^70^

Reasons generated by the victim’s wishes and their interest in privacy do, however, intersect with considerations of solvability, *reducing the presumption to investigate where solvability is low*. Whilst the victim’s strong interest in privacy can be trumped in cases where investigation is necessary and proportionate to prevent significant harm from further sexual offending, the justification for doing this disappears if investigation is not sufficiently likely to achieve the goal of significant harm prevention. This is because an investigation can only be justified if it is both a necessary *and proportionate* means to prevent a greater harm. If solvability is low, the intrusion into the victim’s life is disproportionate because no good is expected to be achieved by the intrusion.

Thus the presumption to investigate, the starting point before considering the remaining morally relevant considerations, is moderated more by solvability in CSA cases than for other offences in which the victim’s interest in privacy is weaker, or the intrusion routinely significantly smaller. Having taken into account the goods that ground the presumption in favor of investigation, and the manner in which this presumption may be tempered by interacting considerations of privacy and solvability, we now turn to the considerations that may weigh against the presumption and those that provide some further reason to investigate.

The most significant consideration when deciding whether to investigate a non-recent CSA offence relates to harm prevention. This is because the most important function of the police is to protect legally enshrined moral rights, including the right to bodily integrity, which is egregiously violated in instances of CSA. Further, the numbers potentially affected by taking steps to prevent future harm add to the significance of this consideration; multiple individuals may be protected through one conviction. More fine-grained assessment of the weight of the reason generated by harm prevention will turn on the estimation of the harm likely to be prevented, including consideration of numbers affected, and the likelihood of achieving this harm prevention. We claim that high offender threat generates a *decisive* reason to investigate; that is, where threat is high, investigation will proceed regardless of other considerations, given the salience of the moral rights at stake. Since failing to prevent future sexual offence is the worst kind of error, low or moderate threat still generates a strong (albeit not decisive reason) to investigate.

The second most significant consideration is the victim’s well-being; specifically, the “rights-like” interests they have in mental and social stability. We claimed that this consideration is secondary to the consideration of harm prevention (specifically, preventing further CSA) because of the nature and degree of this harm, and the possibility of providing compensatory support to the victim to mitigate harm caused to them by the investigation. Although the victim’s interests are significant, and very high levels of victim vulnerability might trump very low likelihood of preventing future sexual assaults through investigation and prosecution, this consideration is nonetheless secondary, for the reasons identified above.

The third most significant consideration is the context of limited resources, and the tradeoffs that pursuing investigation would involve. This consideration generates somewhat weaker reasons than those generated by victim harm, because the negative effects resulting from opportunity costs are less certain and more diffuse than the harm likely to befall a victim, where such harm may be reasonably expected. Limited resources are a derivative consideration pertinent to the goods of fulfilling policing purpose—i.e. harm prevention and doing justice. Limited resources matter because they force us to answer how *best* to fulfill policing purpose *when it is not possible to maximally fulfill it*. High opportunity cost generates a moderate reason not to pursue an investigation. Crucially, this is not just a function of the simple cost of the investigation. Rather, it is a function of the good (in terms of crime prevention and justice) that would be foregone if the costs of the CSA investigation were borne, and so requires consideration of competing priorities and the resources required to achieve fair outcomes.

Legitimacy and societal considerations may generate marginal reasons either way, which might tip the balance once the above considerations have been accommodated. These are ranked fourth because any compromise to public support is less tangible than the harms at stake in the other considerations. Any such compromise is also more uncertain than investigational harms to the victim, and the threat to potential further victims. Further, as outlined above, the will of the community does not need to be fulfilled on every possible occasion in order for the police to be legitimate, and legitimacy does not stand or fall on single decisions (within the plausible range under consideration here).

**Figure 1.**
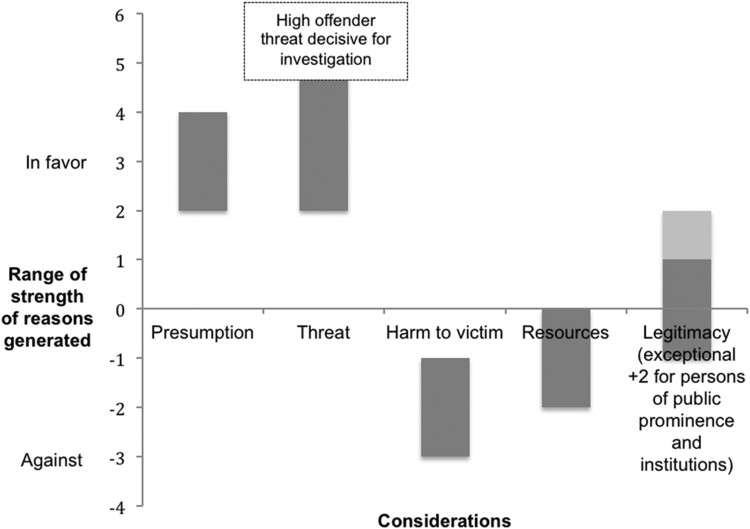
Relative strength of moral reasons generated by the presumption and considerations.

The relative strength of the reasons for and against investigation generated by the presumption and considerations are represented in Figure 1.

Finally, procedural considerations and accountability do not bear on *whether* to investigate but on *how decisions are made and justified*. These considerations include the need to guard against a bias towards making decisions that are less likely to be contested.

### 2. Oxford CSA Framework

We have developed a framework for policing practice. It sets out practical steps for decision-making, which incorporate all theoretical conclusions reached in the section above. It is included in full in the appendix. It uses a numerical approach to structure the weighing up of reasons of differing strength, with room for discretion and required justification. The final decision will involve a process of weighing up the relevant considerations and recognizing that some of the considerations will need to be traded off against one another. This supports a move away from decision-making being a form of artistry within policing, towards a more ethically nuanced and robust decision-making process. However, the final decision will not be arrived at in an unduly mechanical way, and indeed two reasonable decision-makers might arrive at two different conclusions. There remains an important role for discretion, not in a subjective sense, but in something more like practical wisdom that recognizes the diversity of values, and the ways that they need to be integratively understood. This does not, however, undermine the credibility of the approach as providing a useful and principled guide, with room permitted for reasoned rebuttal of the decision indicated by the framework.

### 3. When Considerations Point Away from Investigation

Given the avoidance-of-the-worst-error principle, some might worry that there would be *no* cases in which one could justify not investigating. However, as our framework indicates, the remaining considerations do carry significant weight, and could tip in favor of not conducting an investigation. Table 1 above outlines examples of circumstances that might (depending on the detail) justify not conducting an investigation.

**Table 1:** Features of hypothetical cases and implications for decisions

Features of case	Reasoning and decision indicated
**Case One** Police are made aware of a potential offence against a victim of historical CSA by the local authority. The suspected offender is already in prison for unrelated matters and likely to remain there for many years to come. The potential victim has not approached the police to report the offence, and information suggests that they are mentally vulnerable and at risk of self harm.	In such circumstances, a case could be made out not to approach the potential victim, owing to the low threat posed by the offender, and the high risk of harm to the potential victim.
**Case Two** Police are made aware of a potential historical CSE case by the local authority; 20 years ago children regularly went missing from a care home, had numerous older “boyfriends”, and unexplained gifts and money. The case involves potentially many victims and dozens of offenders; some of whom are deceased, others in prison on unrelated matters, while others appear to no longer be offending. None of the potential victims have complained to the police.	Conducting an investigation on this scale is likely to be highly resource intensive and would have significant opportunity costs; an assessment of the resources required estimates that it would take a team of twelve officers working for maybe eighteen months to complete. Given that the threat posed by the potential offenders is low (owing to their being deceased, in prison, or no longer offending) and that the victims have not sought an investigation, a case could be made out not to conduct the investigation.
**Case Three** A report from a third party that a potential victim was subject to abuse twenty years ago as a child. There is no information to suggest vulnerability on the part of the victim, but they have not reported the abuse themselves. The suspect is now confirmed as deceased, and there is no suggestion of the involvement of other offenders.	In such circumstances, a case may be made out not to conduct an unsolicited visit to the potential victim, primarily since the threat posed by the perpetrator is now removed, and the potential to harm the victim remains. However, if the suspect were a person of public prominence such that there would be a significant impact on public confidence if the police did not conduct an investigation, then a case could be made out to investigate.
**Case Four** Information comes into the possession of the police via intelligence that there was historical CSA against a victim who has not reported it to the police. The potential victim has previously engaged in self-harm and is receiving support from the local authority. The potential offender continues to have access to children through working at a youth group and has a number of other low-level convictions for acquisitive crime.	In such circumstances, despite the risk of harm to the potential victim from an unsolicited police approach, the threat posed by the potential offender is such that it outweighs this risk. However, compensatory support must be provided to mitigate harm to the victim.

## VII. Conclusion

We have developed a principled framework for making decisions about whether to investigate non-recent CSA complaints. We have identified the most significant considerations and argued for their relative weight. The framework that we have presented allows multiple considerations to bear on the decision, considerations that generate reasons for or against the decision, exerting weight regardless of their position in the sequence of considerations. We have argued that this way of proceeding provides the most justified estimate of the decision that should be made. We emphasize, however, that room for rebutting the decision indicated by the framework should be retained, where the decision-maker identifies relevant features of the particular case that are not captured by the high-level considerations. Lastly, we suggest that our approach to generating a framework could be used as a model for decision-making within policing beyond the investigation of non-recent CSA.

## Notes

1 See Grierson, “Police Child Abuse Inquiries.”

2 See “Saville Abuse Part.”

3 See “Westminster ‘Paedophile Ring’ Accuser.”

4 See “Operation Conifer.”

5 See “Hundreds of Telford Child.”

6 See “Rotherham Child Abuse.”

7 See “Rochedale Grooming.”

8 See “Oxfordshire Grooming Victims.”

9 Operation Hydrant is the co-ordination of British police investigations into complaints of “non-recent” child sexual abuse, particularly by high profile public figures or that has taken place within institutions.

10 See Office for National Statistics, *Abuse during Childhood*.

11 Data derived from Home Office Data Hub (HODH). Acessed November 23, 2018. https://data.police.uk/.

12 See Independent Inquiry into Child Sexual Abuse, *The Impacts of Child Sexual Abuse*.

13 See HM Government, *Working Together to Safeguard Children*.

14 See Miller and Blackler, *Ethical Issues in Policing*; Miller and Gordon, *Investigative Ethics.* In making the will of the community relevant, Miller’s theory incorporates aspects of social contract theories (see e.g. Kleinig, *Ethics of Policing*), according to which policing is justified as a consequence of an explicit or presumed social contract that gives police their mandate.

15 Miller and Blackler, *Ethical Issues in Policing*, 9.

16 Whilst there is an established right to bodily integrity, there is not an equivalent established right to mental integrity. This asymmetry is the subject of lively academic debate. See for example Bublitz and Merkel, “Crimes against Minds.”

17 See Gostin, *Public Health Law*; Pugh and Douglas, “Non-consensual Medical Intervention.”

18 Miller and Blackler, *Ethical Issues in Policing*, 5.

19 See College of Policing, *National Decision Model*.

20 See College of Policing, *The Code of Ethics*.

21 The Nolan principles are the basis of the ethical standards expected of public office holders, including the police. They were first set out by Lord Nolan in 1995 and they are included in the Ministerial code.

22 See College of Policing, *Operation Hydrant SIO Advice* and *Responding to Child Sexual Exploitation*.

23 See Sable et al., “Barriers to Reporting Sexual Assault.”

24 See Weiss, “You Just Don’t Report.”

25 Of course, we should not assume that the “victim” has made any decision at all—a lack of complaint might be due to there having been no offence. Investigation is always needed to confirm initial hypotheses. However, the cases that we are discussing will involve significant amounts of evidence; they will not be opportunistic leads. Contacting potential victims on the basis of little or no intelligence presents risks to the investigation. Trawling is the term given to the process whereby the police contact potential victims, even though they have not been named in the course of the investigation. Current advice is that investigators should avoid contacting potential victims in the absence of firm intelligence owing to the risk that it could give rise to false complaints, a practice that has been heavily criticized in court. (See College of Policing, *Operation Hydrant SIO Advice*.) Nevertheless, the advice does not preclude proactively contacting potential victims on a firm intelligence led basis.

26 A recent case of Thames Valley Police involved an approach to 39 potential victims; 37 did not wish to engage with the police investigation.

27 Data from Thames Valley Police.

28 See Campbell and Raja, “Secondary Victimization of Rape Victims.”

29 See ibid. and Campbell and Raja, “Sexual Assault and Secondary Victimization.”

30 Data from Thames Valley Police.

31 See Youth Justice and Criminal Evidence Act (1999), Section 22A.

32 See College of Policing, *Operation Hydrant SIO Advice*, section 2.4.

33 See Sunshine and Tyler, “Procedural Justice and Legitimacy”; “Moral Solidarity.”

34 See Sentencing Council, *Sexual Offences Definitive Guideline*.

35 See Lussier and Healey, “Developmental Origins of Sexual Violence.”

36 See Hanson and Morton-Bourgon, *Predictors of Sexual Recidivism*.

37 See Mann, Hanson, and Thornton, “Assessing Risk for Sexual Recidivism.”

38 See College of Policing, *Authorised Professional Practice on Risk*, under Principle 1.

39 See Ashworth, *Sentencing and Criminal Justice*, 78–9.

40 See Nagin, *Criminal Deterrence Research*.

41 See e.g. Dolinko, “Mistakes of Retributivism”; Ryberg, “Desert-adjusted Utilitarianism.”

42 See e.g. Feinberg, “Expressive Function of Punishment.”

43 See Ashworth, *Sentencing and Criminal Justice*, 148–51.

44 There is a further possible argument which might point to the possibility not of culpability diminishing over time, but of persons becoming less connected to their earlier selves over time, and therefore becoming less deserving of the punishment that would have been deserved by their earlier self. See Parfit, *Reasons and Persons*, 325–26, for a version of this view; Dresser, “Personal Identity and Punishment” for discussion, and Buchanan, “Advance Directives,” 292–94, for parallel discussion in medicine. Such an argument would require that the individual either lacks sufficient access to memories (particularly of their offending) or has changed in significant ways. We do not have space to discuss this argument here, although we do not find it convincing for most CSA cases brought to the police. One exception, however, is the circumstance in which the offender has Alzheimer’s, and is significantly disconnected from their memories and large parts of their character. However, we would argue that since threat is not eliminated, there may be consequentialist grounds on which to investigate, even if there may be implications for severity of punishment at sentencing based on discontinuity of the person.

45 See von Hirsch and Ashworth, *Proportionate Sentencing*, 178. They argue that “with the lapse of time, the possibility increases that the actor may have changed significantly—so that his long past act does not reflect badly on the person he now is.”

46 See National Centre for Policing Excellence, *Murder Investigation Manual*.

47 See Smith, “They Think They’ve Got Away.”

48 See “Jimmy Savile Scandal.”

49 See Dearden, “Rotherham Grooming Gangs.”

50 See Paine and Majchrzak, “Managing Complexity.”

51 A complex CSE investigation is defined as being one that cannot be investigated by a single investigator working alone and that will usually require the skills of a Senior Investigating Officer (SIO).

52 See Home Office, *Statistical Bulletin*, 31.

53 See “Core Policing Under Pressure.”

54 HMICFRS, *State of Policing*.

55 See Paine and Majchrzak, “Managing Complexity.”

56 See Hough, “Modernisation and Public Opinion”; Jackson and Sunshine, “Public Confidence in Policing.”

57 See Sunshine and Tyler, “Procedural Justice and Legitimacy”; “Moral Solidarity.”

58 See “Inquiry Call Over Telford.”

59 See “Lord Bramall.”

60 See “Metropolitan Police ‘Regrets’.”

61 See “Cliff Richard.”

62 See “Westminster ‘Paedophile Ring’ Accuser.”

63 See Scott, “There is No Evidence.”

64 See the IOPC’s webpages at https://www.policeconduct.gov.uk/.

65 See “David Duckenfield.”

66 See Reuss-Ianni and Ianni, “Street Cops and Management Cops.”

67 See College of Policing, *Authorised Professional Practice on Risk*, under Principle 4.

68 See Chief Constable of the Hertfordshire Police v. Van Colle [2008] UKHL 50.

69 Decisions in these cases should normally be made on a victim-by-victim level rather than at the level of the overall investigation, since this will enable calibration of the weighting for each reason to the finest level of morally-relevant detail. However, this position will often be complicated by the fact that victims will not always be independent; there may be some degree of interdependence of events and evidence. This can result in dilemas if some victims are more vulnerable than others. In such cases the avoidance-of-the-worst-error principle should lead investigators to chose the approach based on avoiding harm to the most vulnerable of the victims being considered.

70 For further discussion of this position in the context of domestic violence, see Hanna, “No Right to Choose.”
